# Why we need to integrate mental health into pandemic planning

**DOI:** 10.1177/1757913920957365

**Published:** 2020-10-17

**Authors:** CR Brewin, J DePierro, P Pirard, C Vazquez, R Williams

**Affiliations:** University College London, Gower Street, London WC1E 6BT, UK; Icahn School of Medicine at Mount Sinai, New York, NY, USA; Agence Nationale de santé Publique, Saint-Maurice, France; Universidad Complutense de Madrid, Madrid, Spain; University of South Wales, Pontypridd, UK

*In this article, Chris Brewin and colleagues draw on their experiences of managing the mental health consequences of major incidents, including in the case of pandemics, and highlight how responses in this area tend to be inadequately planned and funded.*

Since the outbreak of COVID-19, the extraordinary pressures placed on many healthcare staff who are dealing with the crisis have rapidly become obvious. Staff face relentless demands, lack of resources, involvement in very difficult clinical decisions, and severe risk to themselves and their families.^1^ The more limited SARS epidemic of 2002–2003 demonstrated that significant consequences, primarily distress in the psychosocial domain and posttraumatic stress disorder and depression in the mental health arena, are to be expected for a substantial proportion of staff and also for those survivors who require intervention, assessment and treatment.^2,3^ The economic impact will exert its own separate toll on nations’ mental health.^4^ Many members of the public are likely to develop distress, depression and prolonged grief by isolation, loss of income and losing family members in heart-breaking circumstances with the possibility of comforting rituals drastically curtailed.^5^

World Health Organization guidance on pandemic influenza risk management^6^ includes as one of the possible necessary responses ‘Address the psychological impacts of the pandemic, especially on the health workforce, and provide social and psychological support for health care workers, patients and communities’. Yet, apart from some consideration of patients with existing psychiatric conditions, there is virtually no mention of mental health consequences in official UK documents such as the 2012 Health and Social Care Influenza Pandemic Preparedness and Response,^7^ or the London 2018 ‘Pandemic Influenza Framework’.^8^

Within the US, expert-panel guidance on psychosocial and mental health needs related to public health emergencies including pandemics^9,10^ is not reflected in the 2014 US Preparedness and Response Framework for Influenza Pandemics report.^11^ Other countries make little if any mention of the need for psychosocial preparedness and intervention (e.g. France: ‘Plan national de prévention et de lutte ‘Pandémie grippale’ (2011); Germany: ‘Nationaler Pandemieplan’ (2017); Spain: ‘Plan de la Pandemia de Gripe’ (2005–2006).^12^

In practice, a variety of ad hoc initiatives to address people’s psychosocial and mental health needs have been speedily instituted in all these countries but the frequent absence of integration within the entire response framework, or of a responsible authority being previously identified to oversee them, has led to multiple negative consequences. One is that national and local responses are being developed with few formal mechanisms for cooperation, leading to duplication of effort and inconsistency in the content and distribution of messaging conveyed to staff and the public. As a result, we hear reports of health services being inundated with well-meaning but ad hoc advice that they must find difficult to evaluate. Another is that care pathways are having to be developed from scratch in the absence of agreements about key components such as: funding; models of care, assessment, and treatment; organisation and integration between statutory healthcare and public health and third sector agencies; and data collection, sharing and governance. Previous experience with major incidents in the UK has repeatedly demonstrated that existing funding and data sharing arrangements have blocked the rapid deployment of psychosocial and mental healthcare pathways and led to enormous inefficiency.^13,14^ Furthermore, in countries such as the US, loss of health benefits coverage due to pandemic-related unemployment is likely to limit access to care; the absence of a unified safety net in disaster response plans for these in-need groups is of particular concern.

Clinical knowledge of how to protect people’s mental health following major incidents is well-advanced. It involves a coordinated suite of interventions that are likely to include general information and advice and short-, medium- and longer-term psychosocial support in various forms. In the longer-term, some form of outreach and screening is frequently required to identify people who need formal mental health interventions but who will not otherwise receive treatment.^15^ These initiatives, along with financial advice and support packages appropriate to the nature of the incident, are necessary to avoid the potential for long-term disruption to health and economic productivity, and the increased risk of stress-related disorders such as cardiovascular disease.

Some national initiatives partly meet these needs, for example, the organisation in France of emergency psychosocial support teams to attend to victims in response to exceptional health situations.^16^ But most of the time, mental health has not enjoyed the sophisticated planning and governance arrangements that guide emergency interventions for physical injury and infections. We call therefore for international resolve to learn the lessons of COVID-19. Public health systems should create national units responsible for maintaining and updating the organisational and scientific knowledge base and fully integrating mental health into thinking and planning for all future major incidents. Funders should plan to find the substantial additional finance that will be required to meet the mental health needs following such incidents.

## References

[1] Greenberg N Docherty M Gnanapragasam S, et al Managing mental health challenges faced by healthcare workers during covid-19 pandemic. Br Med J 2020;368:m1211. 32217624 10.1136/bmj.m1211

[2] Maunder RG Lancee WJ Balderson KE, et al Long-term psychological and occupational effects of providing hospital healthcare during SARS outbreak. Emerg Infect Dis 2006;12(12):1924–32 10.3201/eid1212.060584 PMC3291360 17326946

[3] Mak IWC Chu CM Pan PC, et al Long-term psychiatric morbidities among SARS survivors. Gen Hosp Psych 2009;31(4):318–26. 10.1016/j.genhosppsych.2009.03.001 PMC7112501 19555791

[4] Chaves C Castellanos T Abrams M, et al The impact of economic recessions on depression and individual and social well-being: the case of Spain (2006-2013). Soc Psychiatry Psychiatr Epidemiol 2018;53(9):977–86. 10.1007/s00127-018-1558-2 29992341

[5] Adhanom Ghebreyesus T Addressing mental health needs: an integral part of COVID-19 response. World Psych 2020;19(2):129–30. 10.1002/wps.20768 PMC7214944 32394569

[6] World Health Organization (WHO). Pandemic influenza risk management: a WHO guide to inform and harmonize national and international pandemic preparedness and response. Geneva: WHO, 2017.

[7] Department of Health. Health and social care influenza pandemic preparedness and response, https://assets.publishing.service.gov.uk/government/uploads/system/uploads/attachment_data/file/213696/dh_133656.pdf (2012, accessed 10 September 2020).

[8] London Resilience Partnership. Pandemic influenza framework, https://www.london.gov.uk/sites/default/files/london_pan_flu_framework_v7_may_2018_0.pdf (2018, accessed 10 September 2020).

[9] Pfefferbaum B Flynn BW Schonfeld D, et al The integration of mental and behavioral health into disaster preparedness, response, and recovery. Disaster Med Public Health Prep 2012;6(1):60–6. 10.1001/dmp.2012.1 22490938

[10] Reissman DB Watson PJ Klomp RW, et al Pandemic influenza preparedness: adaptive responses to an evolving challenge. J Homeland Sec Emerg Manage 2006;3(2):13.

[11] Centers for Disease Control and Prevention. Updated preparedness and response framework for influenza pandemics, https://www.cdc.gov/flu/pandemic-resources/pdf/mmwr-rr6306.pdf (2014, accessed 10 September 2020).

[12] European Centre for Disease Prevention and Control, https://www.ecdc.europa.eu/en/seasonal-influenza/influenza-pandemic-preparedness (accessed 10 September 2020).

[13] Cyhlarova E Knapp M Mays N. Responding to the mental health consequences of the 2015-2016 terrorist attacks in Tunisia, Paris and Brussels: implementation and treatment experiences in the United Kingdom. J Health Serv Res Pol 2020;25:172–80. 10.1177/1355819619878756 31769712

[14] Allsopp K Brewin CR Barrett A, et al Responding to mental health needs after terror attacks. Br Med J 2019;366:l4828. 31409609 10.1136/bmj.l4828

[15] Williams R Bisson JI Kemp V Health care planning for community disaster care. In: Ursano RJ Fullerton CS Weisaeth L, et al (eds.) Textbook of disaster psychiatry. 2nd edn. Cambridge: Cambridge University Press; 2017, pp. 244–60.

[16] Philippe J-M Brahic O Carli P, et al French Ministry of Health’s response to Paris attacks of 13 November 2015. Crit Care 2016;20:85. 27039082 10.1186/s13054-016-1259-8 PMC4818525

